# Impact of deep resection of endometriosis in the pelvis on urodynamic parameters

**DOI:** 10.1590/acb386323

**Published:** 2023-12-01

**Authors:** Jardel Cavalcante de Farias, Maria do Desterro Soares Brandão Nascimento, Plínio da Cunha Leal, Caio Márcio Barros de Oliveira, Ed Carlos Rey Moura

**Affiliations:** 1 Universidade Federal do Maranhão – Postgraduate Program in Adult Health – São Luís (MA) – Brazil.

**Keywords:** Endometriosis, Laparoscopy, Urogenital System, Urodynamics, Quality of Life

## Abstract

**Purpose:**

To evaluate the effects of deep resection of endometriosis in the posterior pelvic region on urodynamic parameters.

**Methods:**

A prospective observational study conducted with female patients diagnosed with deep pelvic endometriosis before and after endometriosis resection surgery. Clinical history, image exams, the Female Lower Urinary Tract Symptoms questionnaire, urodynamic examination, cystometry, and voiding study were evaluated.

**Results:**

Patients aged 30–39 years old, operative duration of 132.5 minutes, and 2.7 days of hospital stay. Uroflowmetry and cystometry showed tendency for an increase after the surgery in the flow duration, time to maximum flow, and first voiding desire and decreased residual volume and maximum cystometric capacity. Opening, maximum urinary flow, and maximum flow pressure decreased at T1, and the closing parameters increased, although statistically non significant. The variables decreased at T1 in the urodynamic, except for detrusor overactivity. Although we observed a reasonable number of low bladder compliance and abnormal bladder sensation, the results were maintained at T1. General scores for filling and incontinence showed a significant decrease after surgery.

**Conclusions:**

A significant response in the patient’s perception of urinary function was demonstrated after surgery. It is observed that the surgical procedure did not affect the uroflowmetric and cystometric characteristics of the evaluated patients.

## Introduction

Endometriosis is a disease characterized by endometrial tissue outside the uterine cavity. It affects 10% of women of reproductive age and it is present in 20 to 40% of women with infertility^1^. The most common symptoms are dysmenorrhea, chronic pelvic pain, and dyspareunia^2^.

Endometriosis may affect ovaries, bladder, and ureters. The posterior pelvic compartment is affected when the disease is found in the uterosacral ligaments, posterior vaginal wall, vaginal septum, and rectum^3^. Treatment can be medicinal, surgical, or combined, depending on the severity of symptoms, extent, location of the tissue, desire for pregnancy, age, and cost^4^.

Laparoscopy is the surgical technique of the first choice treatment of the disease. It has proven effective and allows the removal of lesions by fulguration, coagulation, vaporization, or excision, with advantages in postoperative recovery time, blood loss, postoperative pain, and hospital stay^4^. Postoperative complications include changes in urinary function, mainly due to iatrogenic injury^5^. The common pelvic nerves in resecting endometriotic lesions involve the superior hypogastric plexus, splanchnic nerves, inferior hypogastric plexus, and drainage pathways. These nerves are mainly identified in the dissected lateral, medial, retro-rectal, and pararectal spaces^6^. Nerve-sparing surgical techniques reduce the risk of urinary function complications and result in higher postoperative satisfaction.

This study investigated the effects of the laparoscopic approach for deep resection of endometriotic lesions in the uterosacral ligaments and rectovaginal septum on preoperative and postoperative urodynamic parameters.

## Methods

This prospective and observational study was conducted with female patients diagnosed with deep posterior pelvic endometriosis between January 2019 and January 2021.

Females over 18 years old diagnosed with endometriosis based on symptoms, clinical examination, ultrasonography, and magnetic resonance imaging confirmed by histopathologic examination were included in the study. Patients with pain syndromes in the central and peripheral nervous systems and taking antidepressants, antihypertensive drugs, and pharmacological treatments for lower urinary tract disorders were excluded. The patients’ data from this study was obtained as continuous sampling during the collection period.

The patients were evaluated from clinical history, physical examination, and image exams with an indication for surgical treatment previously selected for the study. Among them, only patients diagnosed with endometriosis in the pelvis floor, with injuries limited to the uterosacral and septum ligaments, were included. We defined T0 as the time of examinations before surgery and T1 as the time of reexamination (three months) after surgery.

The evaluation of cystometry refers to the part of the urodynamic study that involves bladder filling. The maximum cystometric capacity, vesical compliance, detrusor sensitivity, detrusor activity, and urinary loss were evaluated through it. After asepsis, two urethral tubes (numbers 6 and 8) and a rectal tube were introduced, proceeding with the bladder filling with 0.9% saline solution.

The voiding study refers to urodynamics that assesses pressure at maximum flow, bladder pressure, voided volume, and post-void residual volume. After reaching the maximum cystometric capacity in cystometry, the patient is invited to sit and urinate spontaneously with the probes present. After the end of urination, the post-micturition residue is measured.

The patients were evaluated for the International Consultation on Incontinence Modular Questionnaire on Female Lower Urinary Tract Symptoms (ICIQ-FLUTS). The questionnaire focuses on the extent of the patient’s symptoms, including urinary incontinence, impact on sexual function, and quality of life, and it is sensitive to changes in female symptoms^7^.

Angelo et al.^8^ emphasized as ideal for monitoring treatment outcomes, as they are derived from the Bristol Female Lower Urinary Tract Symptoms Questionnaire. The assessment is performed considering three domains:

- F (Filling): symptoms related to the filling phase of the bladder, such as nocturia (2A), urinary urgency (3A), bladder pain (4A), and frequency (5A);
- V (voiding): includes the symptoms of the bladder voiding phase, hesitation (6A), effort to urinate (7A), and interruption (8A);
- I (incontinence): refers to the types of urge urinary incontinence (9A), effort (10A), incontinence without apparent reason (11A), and nocturnal enuresis (12A), as well as the frequency of urinary leakage (13A).

The surgical technique used is based on the laparoscopic approach with nerve preservation^9^, which aims to identify the nerve structures during surgery, and this requires advanced laparoscopic skills and accurate anatomic and neuroanatomic knowledge, leading to the isolation and removal of all affected tissue with appropriate surgical radicality, releasing the somatic and autonomic nerves. In the selected cases, only foci in the posterior compartment were resected. They were defined with the revised American Society for Reproductive Medicine (rASRM) scale, the most used staging of endometriosis^10^, which considers the size, depth, and location of endometriotic implants and the severity of adhesions.

The surgeries took place at a specialized center in the follow-up of patients with endometriosis and were performed by the same surgeon, who has 10 years of experience in the surgical treatment of deep endometriosis.

The study was conducted following the guidelines of the Declaration of Helsinki, approved by the Ethics Committee of the Universidade Federal do Maranhão (Protocol No. 23523.006778/2019-47). All patients signed the free and informed consent form.

Data were analyzed using the Statistical Package for the Social Sciences 21.0 program. Normality was tested using the Shapiro-Wilk’s test. The paired t-test or Wilcoxon’s test was applied to compare numerical variables before and after the surgery, and, for categorical variables, the McNemar’s test was performed. P was considered significant when p < 0.05.

## Results

Among the 33 women evaluated, most were aged between 30 and 39 (48.5%), with an overall mean of 37 years old, nulliparous (60.6%), all complained of dysmenorrhea (100%), with an overall mean score of visual analog scale (VAS) of 8.5, 72.7% reported dyspareunia, and 69.7% had not undergone previous pelvic surgery. The mean duration of the procedure was 132.5 (±54.7) minutes, and the hospital stay was 2.7 (±1.0) days, with no recurrence or complications related to the procedure (Table 1).

**Table 1:** Sociodemographic and clinical characterization of women attended to at a gynecological hospital for endometriosis. São Luís, Maranhão, Brazil, 2021.

Variables		n (%)
Age (years old)		
	20–29	3 (9.1)
	30–39	16 (48.5)
	40–48	14 (42.4)
	MD±DP	37.0±7.4
Number of Children		
	0	20 (60.6)
	1	6 (18.2)
	2	3 (9.1)
	3	4 (12.1)
	BMI	23.8±2.6
Dysmenorrhea		
	Yes	33 (100)
	No	0 (0)
	VAS Dysmenorrhea	8.5±1.1
Dyspareunia		
	Yes	24 (72.7)
	No	9 (27.3)
VAS Dyspareunia		8.8±0.6
Surgery time (min)		132.5±54.7
Length of stay (days)		2.7±1
Surgical complications		
	No	33 (100)
Surgical reapproach		
	No	33 (100)
Previous gynecological surgeries		
	None	23 (69.7)
	Cesarean	6 (18.2)
	Oophorectomy	1 (3)
	Oophoroplasty	2 (6.1)
	Salpingectomy	1 (3)
Surgical complications		
	No	33 (100)
Surgical reapproach		
	No	33 (100)
	Total	33 (100)

MD±SD: mean±standard deviation; BMI: body mass index; VAS: visual analog scale. Source: Elaborated by the authors.

Uroflowmetry and cystometry study showed no statistically significant differences between time points T0 and T1, but a tendency for an increase after the surgical procedure in the flow duration, time to maximum flow, and first voiding desire (mL) was observed. A decrease in mean values was also observed concerning residual volume (mL) and maximum cystometric capacity (MCC) (Table 2).

**Table 2:** Pre- and postoperative uroflowmetry (T0 and T1) of women undergoing endometriosis resection treated at a gynecological hospital. São Luís, Maranhão, Brazil, 2021.

Variables		T0	T1	p-value
		Med	Med	
		(min–max)*	(min–max)*	
Uroflowmetry				
	Flow duration(s) (MD±SD)	62.7±24.8	67.9±24.6	0.429 §
	Time to maximum flow(s)	16 (8–68)	20 (7–69)	0.507 £
	Maximum flow (mL)	16 (5–27)	15 (7–42)	0.800 £
	Average flow (mL)	8 (4–15)	8 (4–15)	0.481 £
	Urinary volume (mL) (MD±SD)	421.9±119.5	420.1±91.2	0.936 §
Cystometry				
	First desire to void (mL)	128 (40–300)	170 (40–380)	0.137 £
	Residual volume (mL)	30 (0–230)	10 (0–175)	0.066 £
	MC^2^ (mL) (Md±DP)	475.9±110	458.4±83.3	0.344 §

MD±SD: mean±standard deviation;

* median (minimum–maximum);

T0: preoperative; T1: postoperative; § paired t test; £ Wilcoxon; MC: maximum cystometric capacity. Source: Elaborated by the authors.

Although there were no significant differences between time points in the study of pressure parameters, the variables opening pressure, maximum pressure, and maximum flow showed a decrease in mean values at time T1. Furthermore, closing showed an increase in the mean (Table 3).

**Table 3:** Study of pressure (PDet) of pre- and postoperative urodynamic parameters (T0 and T1) of women undergoing endometriosis resection treated at a gynecological hospital. São Luís, Maranhão, Brazil, 2021.

Pressure study	T0	T1	p-value
	Med (min–max)*	Med (min–max)*	
MCC (cmH2O)	15 (1–30)	15 (6–44)	0.917 £
Opening (cmH2O) (MD±DP)	22.7±13.4	18.9±10	0.188 §
Maximum urinary flow (cmH2O) (MD±DP)	39.1±14.6	34.6±9.1	0.100 §
Maximum flow (cmH2O) (MD±DP)	39.4±17.7	34.7±12	0.218 §
Closing (cmH2O)	28 (4–70)	31 (0–55)	0.712 £

MC: maximum cystometric capacity;

* median (minimum–maximum);

T0: preoperative; T1: postoperative; § paired t test; £ Wilcoxon; MD±SD: mean±standard deviation. Source: Elaborated by the authors.

No significant differences were noticed in the urodynamic observations between time points. The variables showed a decrease at T1, except for detrusor overactivity. Although we observed a reasonable number of low bladder compliance and abnormal bladder sensation, the results were maintained at time T1 (Table 4).

**Table 4:** Presence of pre- and postoperative urodynamic observations (T0 and T1) of women undergoing endometriosis resection treated at a gynecological hospital. São Luís, Maranhão, Brazil, 2021.

Urodynamic observations	T0	T1	p-value €
	n (%)	n (%)	
Low bladder compliance^a^	15 (45.5)	19 (57.6)	1.000
Abnormal bladder sensation^b^	12 (36.4)	8 (24.2)	0.687
Detrusor hyperactivity^c^	0 (0)	0 (0)	Na
Abnormal residual urine^d^	8 (24.2)	4 (12.1)	0.727
Low maximum cystometric capacity^e^	2 (6.1)	1 (3)	0.500

a Bladder compliance (urine volume + residual volume ÷ maximum cystometric capacity) < 30 mL/cmH2O;

b first desire to void occurs in cystometry < 80 or > 200 mL;

c involuntary detrusor contractions during the filling phase;

d post-voiding residue > 100 mL;

e maximum cystometric capacity < 300 mL; Na: absence of 2x2 crossing to perform the test; T0: preoperative; T1: postoperative;

€ McNemar. Source: Elaborated by the authors.

Urinary function decreased values in several parameters (filling, voiding, and incontinence) after surgery, with a significant difference between time points T0 and T1. Nocturia showed no difference in the filling score between the times evaluated, but there was a significant difference in the general score (Table 5).

**Table 5:** Pre- and postoperative ICIQ-FLUTS score (T0 and T1) of women undergoing endometriosis resection treated at a gynecological hospital. São Luís, Maranhão, Brazil, 2021.

Score ICIQ-FLUTS	T0	T1	p-value £
	Med	Med	
	(min–max)*	(min-max)*	
*Filling (Score F)*	6 (1–16)	2 (0–9)	< 0.001
Nocturia	2 (0–4)	1 (0–4)	0.034
Urgency	0 (0–4)	0 (0–2)	0.004
Bladder pain	2 (0–4)	0 (0–4)	< 0.001
Frequency	1 (0–5)	0 (0–3)	0.002
*Voiding (Score V)*	1 (0–12)	0 (0–10)	0.077
Hesitation	0 (0–4)	0 (0–2)	0.020
Stress	0 (0–4)	0 (0–4)	0.168
Intermittency	0 (0–4)	0 (0–4)	0.037
*Incontinence (Score I)*	1 (0–11)	0 (0–4)	0.002
Urge Incontinence	0 (0–3)	0 (0–2)	0.014
Frequency of urinary incontinence	0 (0–4)	0 (0–2)	0.010
Stress urinary incontinence	0 (0–3)	0 (0–2)	0.010
Unexplained urinary incontinence	0 (0–3)	0 (0–1)	0.021
Nocturnal enuresis	0 (0–2)	0 (0–2)	0.564

* Median (minimum–maximum); T0: preoperative; T1: postoperative; £ Wilcoxon. Source: Elaborated by the authors.

## Discussion

This prospective and observational study aimed to investigate the functional effects of endometriosis on the nerve plexuses responsible for bladder sensitivity and motricity and the possible impact of surgical resection on this function. It was observed that there is a significant improvement in lower urinary tract symptoms perceived by the evaluated women.

The fourth decade of life is the most commonly encountered period in female patients at the surgery, as shown here and in other studies^11^. The higher frequency of nulliparous patients could lead to an association with infertility, but we do not have the necessary data to make this statement. Several similar studies showed that the main symptom was dysmenorrhea, followed by dyspareunia. These are the main indications for surgical treatment.

There were no cases of complications such as infections, fistulas, or repeat surgical procedures, which can be explained by factors such as surgical technique, age, weight, lack of previous surgery, short operative duration, and short hospital stay, as observed in the study by Ceccaroni et al.^12^. In addition, all surgeries were performed by the same surgical team, which probably provides a higher degree of uniformity concerning the surgical procedure.

There were no uroflowmetric and cystometric differences between time points T0 and T1, similar to what was shown by Ballester et al.^13^. This was also observed in the pressure difference in the variables of opening pressure, maximum urinary flow, and maximum flow. In some situations, there is a tendency to have differences, but it should be noted that both pre- and postoperative were within the normal range for the clinical interpretation.

Urodynamic observations show that the frequency of low bladder compliance increases in the postoperative period. These findings are also commonly found with direct bladder involvement, including muscle layer injury and the need for partial bladder resection, as demonstrated by Resende Junior^14^. However, direct bladder injury was an exclusion factor for patient selection in our evaluation. This opens a line for future evaluation of bladder control in cases of direct injuries with and without nerve involvement.

Also noteworthy is the high frequency with which abnormal bladder sensation was noted before intervention (T0), in which 36.4% identified the early first desire to void occurs in cystometry. Theoretically, it could harm function, which showed improvement after surgery, significantly reducing the number of cases. In clinical practice, complaints of shortening the voiding urge interval among women with deep endometriosis are a constant. Although we still do not have an explanation of the exact mechanism involved, we agree that the urodynamic study may suggest a way to get this answer. However, the presence of the disease and its characteristic inflammatory environment near the nerves affect the pattern of nerve conduction, so there are well-established patterns in inflammatory diseases and their mechanisms of influencing nerve function.

Serati et al.^15^ and Spagnolo et al.^16^ found a high percentage of overactive bladder, 90 and 40%, respectively, in patients with endometriosis before laparoscopy. The involvement of the nervous plexuses in the parametrium has been described as an influencing factor on bladder function^17^, directly impacting the urodynamic parameters, such as the overactive bladder. In contrast, no overactive bladder was detected in the present study. However, it should be noted that we also excluded cases of endometriosis with bilateral parametrial involvement from our evaluation, which may even corroborate their data.

Some authors reported an association between increased ICIQ-FLUTS scores and deep endometriosis^18^. For this connection, other authors have demonstrated a change in the pattern and distribution of nerve fibers in the abdomen and the inflammatory stimulus near the nerves, possibly causing this association^19^. In the present study, the questionnaire ICIQ-FLUTS showed high scores in patients with endometriosis filling and voiding, followed by a decrease after surgery. The marked decrease in scores suggests an overall improvement in patients’ lower urinary tract-related symptoms.

As in the present study, Bonneau et al.^20^ also described improving urinary symptoms after surgery to treat deep endometriosis of the posterior compartment. In this sense, we emphasize that reducing the inflammatory environment caused by the resection of endometriosis foci may influence nervous physiology and directly affect bladder function. Furthermore, the improvement in urinary function perceived by the patients, as shown by the results of the questionnaires, may confirm this hypothesis.

Although the study did not demonstrate statistically significant differences in urodynamic parameters, the examination is still considered an option in various clinical situations, such as patients with suspected neuropathic pelvic pain, a history of surgery for endometriosis, the urinary tract symptoms improved post-operatively. Therefore, this study may serve as a model for developing protocols to assess urinary function before and after endometriosis surgery to investigate possible prior impairment or verify that surgery has not affected urinary function.

As a limitation, the control group was not used for ethical reasons. The impossibility of using a control group is also since patients not selected for surgery would probably be on hormone therapy. The follow-up period was also limited (three months), as some studies showed a better response in several parameters, especially bladder motility, at six months after surgery.

## Conclusion

The surgical procedure and the characteristic nerve resection did not affect the evaluated patients’ uroflowmetry and cystometry.

## Data Availability

The data will be available upon request.
